# Influence of genetic abnormalities on semen quality and male fertility: A four-year prospective study

**Published:** 2014-02

**Authors:** Fadlalla Elfateh, Ruixue Wang, Zhihong Zhang, Yuting Jiang, Shuang Chen, Ruizhi Liu

**Affiliations:** *Reproductive Medical Center, First Hospital of Jilin University, Changchun, Jilin, China. *

**Keywords:** *Chromosomal abnormalities*, *Male infertility*, *Semen quality*, *Y -chromosome microdeletions*

## Abstract

**Background:** Wide range of disorders ranging from genetic disorders to coital difficulties can influence male fertility. In this regard, genetic factors are highlighted as the most frequent, contributed to 10-15%, of male infertility causes.

**Objective: **To investigate the influence of genetic abnormalities on semen quality and reproductive hormone levels of infertile men from Northeast China.

**Materials and Methods:** 2034 infertile men including 691 patients with abnormal sperm parameters were investigated retrospectively. Semen analysis was performed according to the World Health Organization guidelines. Y chromosome micro deletions were detected by polymerase chain reaction assays. Chromosome analysis was performed using G-banding.

**Results:** The incidence of abnormal chromosomal karyotype in the patients with abnormal sperm parameters was 12.01% (83/691). The most frequent cause was Klinefelter's syndrome 37.35% (31/83). As the same as chromosomal abnormalities group, the volumes of testes (p=0.000 and 0.000, respectively) and the levels of testosterone (T) (p=0.000), and testosterone/ luteinizing hormone (T/LH) (p=0.000) of patients with Y chromosome micro deletions were significantly lower than those of fertile group. In addition, the levels of follicle-stimulating hormone (FSH) (p=0.000), and luteinizing hormone (LH) (p=0.000) were significantly higher in patients with Y chromosome micro deletions than those in the fertile group. Translocation abnormalities displayed slight effect on sperm motility.

**Conclusion:** Y chromosome micro deletions and sex chromosome disorders particularly Klinefelter’s (47, XXY), have severe adverse influence on normal hormone levels, testicular volume and sperm count, whereas translocation abnormalities may inversely correlate with sperm motility.

## Introduction

Infertility refers to the biological failure to conceive a child after one year of unprotected intercourse, with the rate of 10-15% worldwide (1). In the past years there was a belief that infertility is a woman’s problem, but in fact, male factors contribute to about half of infertility cases (2). Wide ranges of disorders ranging from genetic abnormalities to coital problems can influence male fertility. Among these genetic factors considered as the most frequent and interesting factors that can adversely affect male fecundity. They were reported at an incidence of 10-15%, in infertile male including chromosomal abnormal karyotype and single gene anomalies (3). 

However genetic disorders that influence male fertility can be classified into two main groups of chromosomal abnormalities and Y chromosome micro deletions. Sex chromosome disorders were dominant in azoospermic and oligospermic infertile men with a prevalence of 4.2%, compared with autosome anomalies which only estimate about 1.5% of both populations (4).

These disorders, in one way or another affect the semen quality and cause various degree of male infertility, by causing disturbance in genomic material which resulting in alteration of chromosome materials. Recently many researches had reported that there is a close relation between genetic disorders and miscarriage, moreover, great association with birth defects has been reported (5). The atrophy of the testis and the decreasing of the sperm count observed in Klinefelter’s syndrome patients may attributed to atresia of the germ cells containing two X chromosome, which theoreticaly results from fatal gene dosage caused by the extra X chromosome (6).

While in translocation, carriers of the translocated segments fail to pair successfully, resulting in free unpaired segment which interfere with the X and Y chromosomes during the first meiotic division. The abnormal pairing during spermatogenesis between the unpaired autosome segments and the X interferes with the normal X inactivation, resulting in a fatal dosage gene impact the germ cells (7). However evaluations of genetics factors that may affect male fertility provide valuable data and theoretical basis for assisted reproductive technologies to solve fertility problems in those patients.

## Materials and methods

This is a prospective study performed during the period from 2008-2012. 

Patients: 2034 infertile men who attend the First, Hospital of Jilin University were first investigated, then the azoospermia patients were first excluded from the study, and then patients with factors that may affect fertility were excluded. These factors include excessive alcohol intake, chronic or hallucinatory drug use, serious systemic disease, abnormality of the external genitalia, known hereditary/familial disorders, and also excluded men involved those who had infection or trauma of the genitals.

Last 691 patients with abnormal sperm parameters were include in our study, in which 327 of 691 patients had normal karyotype, they were tested for Y chromosome microdeletions. Participants in this study were asked to collect their semen samples by masturbation at the hospital in a polypropylene container after 2-7 days of sexual abstinence. The semen were then allowed to liquefy at 37^o^C and processed immediately thereafter using the WHO recommended guideline (8). 

This study was approved by the Reproductive Medicine Ethics Committee of First Hospital of Jilin University, and all patients signed informed consents of this study before semen analysis. 

Abnormal semen status was classified as follows: oligozoospermic (sperm concentration <20×10^6^/mL), severe oligozoospermic (sperm concentration <5 ×10^6^/mL), asthenozoospermic (percentage of a+b grade sperm <50%), teratozoospermic (the percentage of morphologically normal sperm <15%), cryptozoospermic (spermatozoa absent from fresh preparations but observed in a centrifuged pellet), oligoastheonozoospermic (sperm concentration <20×10^6^/mL and percentage of a+b grade sperm <50%), and oligoteratozoospermic (sperm concentration <20×10^6^/mL, and the percentage of morphologically normal sperm <15%). Peripheral blood samples were obtained and stored for cytogenetic detection and hormonal analysis. Another 78 normal fertile men were included as controls. Every man in the control group had fathered at least one child.

**DNA extraction and polymerase chain reaction (PCR) analysis**

Peripheral blood samples were obtained from all patients and the genomic DNA was isolated using commercially-available blood DNA extraction kits (Beijing Tiangen Biotech Co., Ltd, China). Control DNA samples obtained from unrelated normal males with proven fertility and from normal females were used as positive and negative controls, respectively. 

A sample containing all reaction components and water in place of the DNA template was used as the PCR blank control. Based on the recommendations of the European Academy of Andrology (EAA) and the European Molecular Genetics Quality Network (EMQN), the samples were tested for classical Y chromosome micro deletions using the following sequence-tagged sites (STSs) proposed by Simoni *et al*: sY84, sY86, sY127, sY134, sY143, sY152, sY254, sY255, and sY157 (9). 

The detections of sY14 (SRY) and ZFX/ZFY were employed as internal controls. chMultiplex PCR was carried out in a total volume of 30 μL using a Veriti 96-well PCR thermal cycler (Applied Biosystems, USA). The results were considered positive when a clear amplification product of the expected site was obtained. 

**Karyotype analysis**

After culturing peripheral blood samples for 72 h, lymphocyte chromosome spreads were prepared using routine methods; Karyotypes were described according to the International System for Chromosome Nomenclature (ISCN), and analyzed by G-banding (10). For each individual, a minimum of 20 metaphase cells were counted and at least five cells were analyzed. 

**Hormone analysis**

The levels of the reproductive hormone luteinizing hormone (LH), follicle-stimulating hormone (FSH) and testosterone (T), were measured by electrochemiluminescence immunoassays (ECLIA) using the Elecsys 2010 chemistry analyzer (Roche, Germany), based on the manufacturer's instructions. The normal reference ranges for LH, FSH, and T hormones were 1.7-8.6 mIU/mL; 1.5-12.4 mIU/mL; and 2.8-8.0 ng/mL, respectively.

**Statistical analysis**

SPSS software (ver. 11.5; SPSS, Inc., Chicago, IL, USA) was used to perform statistical calculations. Using t-test analysis, and chi-square test. Differences were considered to be statistically significant when p<0.05. 

## Results

Out of studied 691 patients with abnormal sperm parameters, the incidence of abnormal chromosomal karyotype was 12.01% (83/691). These abnormalities include: 31 (one mosaic and 30 non-mosaic) Klinefelter's syndrome 37.35% (31/83), 26 Chromosome polymorphism 31.33% (26/83), 3 cases of 47,XYY 3.6% (3/83), one case of marker chromosome 1.2% (1/83), and 22 (7 Robertsonian and 15 reciprocal) translocation 26.51% (22/83) (Table I and II). As shown in table I and II, out of 31 Klinefelter's syndrome, 24 patients were diagnosed with cryptozoospermia, 6 with severe oligozoospermia and one case with asthenoszoospermia. 

For chromosomal polymorphismic, the results had shown that, out of 26 cases, 14 cases were oligozoospermic, 8 were severely oligozoospermic, 3 were oligoasthenozoospermic, and one was asthenozoospermic. Moreover the study result showed that, out of 3 XYY patients, two were severely oligozoospermic and one was oligoasthenozoospermic. Besides, there was one case of marker chromosome (47, XY, +mar) that was diagnosed with severe oligozoospermia (Table I and II). For translocation, the study results had shown, that, out of 7 Robertsonian translocation patients, 4 were oligoasthenozoospermic, two were oligozoospermic and one was asthenozoospermic. Whereas, of 15 reciprocal translocation cases there were 7 oligozoospermic; 5 oligasthenozoospermic, two severely oligozoospermic, and one asthenoszoospermic case (Table I and III). 

As shown in table IV the testicular volume and reproductive hormone levels of abnormal karyotype patients group were compared with those of normal fertile control group. The results showed that, the volumes of both left and right testis of all abnormal karyotype carriers were significantly lower than those of control group (for the left testis (p=0.000), (p=0.003), (p=0.000), (p=0.000), and (p=0.000), for Robertsonian translocation, Reciprocal translocation, 47,XYY, Chromosome polymorphism ,and klinefelter’s syndrome respectively, (for the right testis (p=0.000), (p=0.001), (p=0.014), (p=0.000), and (p= 0.000) for Robertsonian translocation, Reciprocal translocation, 47,XYY, Chromosome polymorphism ,and klinefelter’s syndrome respectively. 

Further, in all abnormal karyotype carriers the levels of T and T/LH were also significantly lower than those of normal fertile males (for T levels; p=0.000, and for T/LH levels; p=0.000). Moreover, the levels of FSH and LH in Klinefelter’s syndrome, Chromosome polymorphism, and Robertsonian translocation groups, were significantly higher than those in the control group (FSH levels p=0.000), (p=0.000), and (p=0.087) respectively, LH levels (p=0.000), (p=0.002), and (p=0.007) respectively, whereas no significant difference was found in those levels between 47, XYY group and control group. For reciprocal translocation, the levels of LH were significantly higher than those in the control group (p=0.036), whereas no significant difference was noted in the levels of FSH. 

Comparing the testis size and hormonal levels in samples obtained from severe oligozoospermic group, cryptozoospermic group (from men with non-mosaic Klinefelter’s syndrome), and control group, the results showed that, the volumes of both the right and the left testis, and the levels of T and T/LH were significantly lower than those in the control group (p=0.000). On the other hand, the levels of FSH and LH were significantly higher than those in the fertile control group (p=0.000, Moreover, the study results revealed that, volumes of both right and left testis of severe oligozoospermic group (from non-mosaic Klinefelter’s syndrome carriers) were significantly higher than those of cryptozoospermic group (from non-mosaic Klinefelter’s syndrome carriers) (p=0.016 for right testis and p= 0.020 for left testis ); while no significant difference in the levels of hormones was noted between the two groups (Table V).

Regarding motility, the study results noted four cases of abnormal karyotype that showed decrease in sperm motility (asthenozoospermia). These include; one case for each of 45,XY,rob(13;14)(q11;p11), 46,XY,t(7;13)(q36;q22), 47,XXY[5]/46,XY[45], and 46,XY(Yqh-). In addition, nine carriers of translocation and one carrier for each of 46,XY(Yqh-), 47,XYY, 46,XY (22pstk+), and 46,XY,inv(9)(p11q12) were found with oligoasthenozoospermia (Table III).

Regarding Y chromosome micro deletions, the study results found that Y chromosome micro deletions were detected among 38 patients out of 327 patients with abnormal semen parameters, with a prevalence of 11.62% (38/327). Deletion in AZFc region was the most frequent (11.32% (37/327)). The rates of micro deletions were 12.07% (28/232), 10.41% (5/48), 9.52% (2/21), and 33.33% (1/3) for men with oligozoospermia, oligoasthenozoospermia, oligoasthenoteratozoospermia, and oligoteratozoospermia, respectively. Of 48 oligoasthenozoospermic patients, only one patient showed deletion in AZFb region (2.08% (1/48)). No deletion was detected in AZFa region (Table VI). Comparing AZF micro deletions group and the control group in respect to testis volume and hormone levels, the volumes of both right and left testis, and the levels of T and T/LH of AZF micro deletions group were significantly lower than those of control group (p=0.000). Further, the levels of FSH and LH were found to be significantly higher than those in the fertile control group (p=0.000) (Table VII).

**Table I:** Semen status in the carriers of abnormal chromosomal karyotype

	**Oligo-** **zoospermia**	**Severe oligozoospermia**	**Astheno-** **zoospermia**	**Oligo-** **asthenozoospermia**	**Crypto-** **zoospermia**	**Total Patients number**
Robertsonian translocation	2	-	1	4	-	7
Reciprocal translocation	7	2	1	5	-	15
Klinefelter’s syndrome 47,XXY	-	6	-	-	24	30
Klinefelter’s syndrome 47,XXY [5]/ 46,XY[45]	-	-	1	-	-	1
47,XYY	-	2	-	1	-	3
Marker chromosome	-	1	-	-	-	1
Chromosome polymorphism	14	8	1	3	-	26
Total	23	19	4	13	24	83

**Table II:** Karyotype of patients with (Klinefelter’s syndrome, 47, XYY, marker chromosome, chromosome polymorphism) and semen status of them

**Groups**		**Chromosome karyotype**	**Oligo-** **zoospermia**	**Severely oligozoospermia**	**Astheno-** **zoospermia**	**Oligo-** **asthenozoospermia**	**Crypto-** **zoospermia**	**Total Patients number**
Klinefelter’s syndrome								
		47,XXY	-	6	-	-	24	30
		47,XXY[5]/46,XY[45]	-	-	1	-	-	1
47,XYY		47,XYY	-	2	-	1	-	3
Marker chromosome		47,XY,+mar	-	1	-	-	-	1
Chromosome polymorphism								
		46,XY(Yqh+)	3	3	-		-	6
		46,XY(Yqh-)	3	2	1	1	-	7
		46,XY(14p+)	1	-	-	-	-	1
		46,XY(15p-)	1	-	-	-	-	1
		46,XY(21s+)		1	-	-	-	1
		46,XY(21pstk-)	1		-	-	-	1
		46,XY(22s+)	1	-	-	-	-	1
		46,XY(22pstk+)	-	-	-	1	-	1
		46,XY(16qh+)	1	-	-	-	-	1
		46,XY,inv(9)(p11q12)		3	2	-	1	-	6

**Table III:** Karyotype of patients with translocation abnormalities and semen status of them

**Groups**	**Chromosome karyotype**	**Oligo-** **zoospermia**	**Severely oligozoospermia**	**Astheno-** **zoospermia**	**Oligo-** **asthenozoospermia**	**Crypto-** **zoospermia**	**Total Patients number**
Robertsonian translocation							
	45, XY, rob (13;14) (q11;p11)	-	-	1	2	-	3
	45, XY, rob (13;21) (q10;q10)	-	-	-	1	-	1
	45, XY, rob (14;15) (q10;q10)	-	-	-	1	-	1
	45, XY, rob (14;21) (q10;q10)	2	-	-	-	-	2
Reciprocal translocation							
	45, X , der (Y;22) (q10;q10)	-	-	-	1	-	1
	46, XY, t (Y;4) (p11;p14)	-	1	-	-	-	1
	46, XY, t (Y;14) (q11;p11)	1	-	-	-	-	1
	45, XY, rob (15;22) (q10;q10), t (1;11)(q25;q23)	1	-	-	-	-	1
	46, XY, t (1;9) (p22;p24)	-	-	-	1	-	1
	46, XY, t (6;8) (p21;q24)	-	-	-	1	-	1
	46, XY, t (10;13) (q10;q10)	1	-	-	-	-	1
	46,XY,-13,-19,+der(19)t(13;19)(13q12;19p13)	1	-	-	-	-	1
	46, XY, t (4;13) (q12;q12)	-	-	-	1	-	1
	46, XY, t (1;13) (p22;q14)	1	-	-	-	-	1
	46, XY, t (3;12) (q28;q15)	1	-	-	-	-	1
	46, XY, t (6;14) (q13;p10)	-	1	-	-	-	1
	46, XY, t (4;9) (q35;q12)	-	-	-	1	-	1
	46, XY, t (1;2) (q21;p23)	1	-	-	-	-	1
	46, XY, t (7;13) (q36;q22)	-	-	1	-	-	1
Total		9	2	2	9	-	22

**Table IV:** Comparison of age, testis volume, and hormone levels between abnormal karyotype and control group

**Groups**	**Age**	**Left testis volume (ml)**	**Right testis volume (ml)**	**FSH** **(mIU/ml)**	**LH** **(mIU/ml)**	**T** **(ng/ml)**	**T/LH**
Robertsonian translocation (7)	32.86±5.05	11.57±3.82 *	12.14±3.53 *	9.08±8.96 *	8.43±5.65 *	2.86±0.58 *	0.55±0.36 *
P-value	0.223	0.000	0.000	0.087	0.007	0.000	0.000
Reciprocal translocation (15)	29.13±4.02	15.73±3.95 *	15.66±3.98 *	4.57±1.95	5.03±2.52 *	3.49±1.43 *	1.05±0.90 *
P-value	0.379	0.003	0.001	0.242	0.036	0.000	0.000
47,XYY (3)	24.33±3.79*	10.00±3.46 *	10.00±3.46 *	4.02±0.22	3.96±1.50	3.46±0.67 *	0.92±0.17 *
P-value	0.234	0.000	0.014	0.472	0.596	0.000	
Chromosome polymorphism (26)	28.62±4.67	15.00±4.53 *	15.38±4.46 *	12.32±18.28 *	6.41±8.18 *	5.07±4.35 *	1.35±1.79 *
P-value	0.176	0.000	0.000	0.000	0.002	0.000	0.000
KS syndrome (31)	27.84±4.55*	4.77±3.83 *	4.58±3.64 *	32.18±17.99 *	21.68±11.00 *	2.38±1.77 *	0.17±0.21 *
P-value	0.019	0.000	0.000	0.000	0.000	0.000	0.000
Control (78)	30.24±4.77	19.28±3.59	19.79±3.59	4.11±1.89	3.78±1.59	5.30±1.88	1.65±0.88

FSH; follicular stimulating hormone

LH; luteinizing hormone

T; testosterone

T/LH; testosterone/ luteinizing hormone

Comparing with control group, *: p<0.05

**Table V:** Comparison of age, testis volume and hormone level between severe oligozoospermia and cryptozoospermia (in men with non-moisaic Klinefelter’s syndrome and control group

**Groups**	**Age**	**Left testis volume (ml)**	**Right testis volume (ml)**	**FSH** **(mIU/ml)**	**LH** **(mIU/ml)**	**T** **(ng/ml)**	**T/LH**
Severe oligozoospermia (6)	25.33±3.50*	7.33±4.18*#	6.83±4.36*#	32.81±16.87*	21.51±14.35*	2.65±0.91*	0.28±0.37*
P-value (compare control)	0.016	0.000	0.000	0.009	0.029	0.000	0.000
P-value (compare cryptozoospermia)	0.116	0.016	0.020	0.948	0.882	0.585	0.337
Cryptozoospermia (24)	28.63±4.63	3.71±2.80*	3.58±2.47*	33.36±17.77*	22.27±10.26*	2.21±1.88*	0.12±0.13*
P-value (compare control)	0.146	0.000	0.000	0.000	0.000	0.000	0.000
Control (78)	30.24±4.77	19.28±3.59	19.79±3.59	4.11±1.89	3.78±1.59	5.30±1.88	1.65±0.88

Comparing with cryptozoospermia group, # p<0.05

FSH; follicular stimulating hormone

LH; luteinizing hormone

T; testosterone

T/LH; testosterone/ luteinizing hormone

Comparing with control group, *: p<0.05

**Table VI:** AZF andY chromosome microdeletions results of 327 patients with abnormal semen parameters

**Groups**	**Number**	**AZFa deletion** **NO (%)**	**AZFb deletion** **NO (%)**	**AZFc deletion** **NO (%)**	**Total** **NO (%)**
Oligozoospermia	232	0	0	28	28 (12.07)
Asthenozoospermia	18	0	0	1	1 (0.55)
Oligoasthenozoospermia	48	0	1	5	6 (12.5)
Oligoteratozoospermia	3	0	0	1	1 (33.33)
Asthenoteratozoospermia	5	0	0	0	0 (0)
Oligoasthenoteratozoospermia	21	0	0	2	2 (9.52)
Total , No(%)	327	0 (0)	1 (0.31)	37 (11.32)	38 (11.32)

NO; number. (%); percentage

**Table VII:** Comparison of age, testis volume and hormone levels between AZF deletion group and control group

**Groups**	**Age**	**Left testis volume (ml)**	**Right testis volume (ml)**	**FSH** **(mIU/ml)**	**LH** **(mIU/ml)**	**T** **(ng/ml)**	**T/LH**
AZF deletion (38)	29.00±4.47*	13.24±3.72*	13.18±3.97*	13.40±10.09*	6.60±3.46*	3.26±1.43*	0.65±0.47*
Control (78)	30.24±4.77	19.28±3.59	19.79±3.59	4.11±1.89	3.78±1.59	5.30±1.88	1.65±0.88
P-value	0.139	0.000	0.000	0.000	0.000	0.000	0.000

FSH; follicular stimulating hormone

LH; luteinizing hormone

T; testosterone

T/LH; testosterone/ luteinizing hormone

Comparing with control group, *: p<0.05

## Discussion

The present study has been performed to investigate the influence of genetic disorders on semen quality and reproductive hormone levels of infertile men from Northeast China. The most frequent karyotype anomaly found in this study is Klinefelter’s syndrome; these findings are in accordance with those of pervious reports (11-14). All Klinefelter’s syndrome patients showed abnormal testicular size and abnormal levels for FSH, LH, T, and T/LH ratio. Moreover the semen status of the majority if not all of Klinefelter’s syndrome patients was characterized by presence of hardly any viable sperms in the ejaculate. This conclusion is in agreement with those of previous studies (15-17). 

Despite, some studies demonstrated the possibility of overcoming this kind of infertility by using either in-vitro fertilization IVF or intracytoplasmic sperm injection ICSI (18-20). Thus those patients are able to obtain their own sperms by micro dissection testicular sperm extraction rather than donor’s sperm to achieve their own biological child (14, 21-24). In contrast, regarding 47,XYY karyotype carriers, the study results demonstrated that, of three 47,XYY carriers two were severely oligozoospermic and one was oligoasthenozoospermic, all the three patients showed a normal FSH and LH levels. This finding is consistent with conclusions made by Martin in 2008 who reported that, many of the 47,XYY men have normal semen parameters, but frequently showed severe oligozoospermia due to more disturbances that may occur during meiotic division, subsequent loss of germ cells and production of unbalanced sperms (25). 

Our results demonstrated that most infertile translocation carriers were oligozoospermic with measurable amount of sperms in their ejaculate. Thus the infertility of those patients can be treated by assisted reproductive technologies (26). Influence of chromosomal disorders on sperm motility and morphology has not been clearly clarified in most of the previous studies (27, 28). In 2011 a study by Brahem *et al* reported that, for all teratozoospermic patients a normal karyotype and an absence of Y chromosome micro deletion was overt (29). 

These reports do not fully agree with our study where no relation was observed between the abnormal karyotype, and sperm motility in these pervious reports. None-the-less, regarding sperm motility, our study revealed that, chromosomal translocation disorders in addition to their influence on sperm concentration, may relate to the decreasing of sperm motility that half of patients with translocation were diagnosed with either asthenozoospermia or oligoasthenozoospermia. Regarding chromosome polymorphism disorders, the present study described their influence on semen quality as follow: Chromosome polymorphism abnormalities mainly adversely affect sperm concentration, and the semen status of the carriers ranges from oligozoospermia to severe oligozoospermia. Occasionally these disorders may reflect in a negative influence on sperm motility. 

This description is consistent with Teng *et al* who identified the single nucleotide polymorphisms in deleted azoospermic-like region, and specified the single nucleotide polymorphisms which affect sperm concentration and sperm motility (30). Since testicular volume had positive correlation with sperm density, total sperm count, total motile sperm count, and percentage of motile sperms, in addition there is a significant relation between testicular volume and the serum concentration of reproductive hormones (31-35). 

However our results clarified this relationship in all studied genetic abnormalities carriers, where genetic abnormalities of any type is reflected in, the testis volumes, and the levels of T and T/LH ratio that were significantly lower than they that should be. Influence of genetic abnormalities on FSH and LH varies according to the type of abnormalities. The levels were significantly elevated in the majority of abnormalities, except for 47, XYY which was not significantly different from those levels of fertile group. Concerning AZF micro deletions, we found that, deletions in AZFc region was the most frequent. In addition, all patients showed abnormal testicular volumes and hormone levels that was lower than they should be. The majority of patients have various degrees of sperm within the ejaculate. These findings are in agreement with those of previous studies (36-38). 

Furthermore Hopps *et al* had noted that men with micro deletion of the AZFa or AZFb regions of the Y chromosome have very poor amount of sperm, whereas the majority of male with AZFc deletion have sperms within the ejaculate or testes (39). Many studies concluded that male age had a negative correlation with sperm motility; that increasing in male age strongly correlates with decreasing of both normal sperm motility and sperm morphology (40-42). In our results although the patients were significantly younger than the control group, but their semen parameters significantly lower than they should be and then those of fertile control group. This would support the results, and the final conclusion of the result would be efficient. 

The present study concluded that, sex chromosome disorders, particularly 47, XXY, have severe adverse influence on testicular volume and sperm concentration, resulting in lack of viable sperms in the ejaculate, whereas the semen status of patients with autosomal anomalies (translocations) ranges between oligozoospermia to severe oligozoospermia. Genetic disorders affect male fertility by their adverse influence on testicular volume, hormone levels, and sperm concentration. On other hand, although not clear, translocation abnormalities seem to be related to decreasing of sperm motility. However, correlation between translocation abnormalities and sperm motility need to be further explored to reveal possible clinical relation. This will provide proper counseling prior to applying for assisted reproduction techniques.

## Conflict of interest

The authors had no conflict of interests declare in relation to this article.
